# Intraoperative traction does not improve curve correction or pelvic obliquity within a matched cohort of patients with neuromuscular scoliosis

**DOI:** 10.1007/s43390-026-01354-6

**Published:** 2026-04-02

**Authors:** Ian Fletcher, Alastair W. Sterns, Jacquelyn N. Valenzuela-Moss, Tyler A. Tetreault, Tiffany Phan, Efrain Amaro, Gerard K. Williams, Lindsay M. Andras, Michael J. Heffernan

**Affiliations:** 1 Jackie and Gene Autry Orthopedic Center, Children’s Hospital Los Angeles, 4650 Sunset Blvd, Mailstop #69, Los Angeles, CA 90027 USA; 2 Keck School of Medicine of University of Southern California, Los Angeles, CA USA

**Keywords:** Intraoperative traction, Neuromuscular scoliosis, Cerebral palsy, Curve magnitude, Pelvic obliquity, Flexibility index

## Abstract

**Purpose:**

Evaluate the utility of intraoperative traction (IOT) during posterior spinal fusion (PSF) in a matched cohort at a high-volume neuromuscular scoliosis (NMS) center.

**Methods:**

A nested case–control study was performed on a single-center retrospective database of NMS patients. Those diagnosed with cerebral palsy were pair-matched by age (± 1 year), preoperative curve magnitude (± 10°), preoperative traction curve magnitude (± 10°), and flexibility index (± 5%). Paired *t* tests or Wilcoxon signed-rank tests were used to analyze continuous variables between groups. Fisher’s Exact tests were used to compare categorical variables by group.

**Results:**

Thirty-one unique case–control matches were identified (*n* = 62). IOT and non-IOT groups were similar in terms of EBL 855 (IQR 500–1200) vs. 800 (IQR 650–1350) cc (*p* = 0.60), length of ICU stay 1.7 (IQR 1.0–2.7) vs. 2.3 (IQR 1.7–3.1) days (*p* = 0.14), BMI 18.0 (IQR 14–20) vs. 15.5 (IQR 14–18) (*p* = 0.10), sex distribution 54.8% vs. 51.6% (*p* > 0.99), and changes in neuromonitoring signals 4/31 (13%) vs. 3/31 (10%) (*p* > 0.99). Complication rate was 22.6% (7/31) for IOT and 25.8% (8/31) for non-IOT (*p* > 0.999). There were no statistical differences in surgical time 414 ± 131 vs. 397 ± 133 min (*p* = 0.64) or anesthesia time 551 ± 136 vs. 529 ± 135 min (*p* = 0.56). Both groups had similar postoperative curve magnitude IOT = 37° vs. non-IOT = 42° (*p* = 0.28) and percent curve correction IOT = 60% vs. non-IOT = 56% (*p* = 0.30). Percent correction of pelvic obliquity was also similar 78% (IQR 67–90) vs. 68% (IQR 60–91) (*p* = 0.18) between groups.

**Conclusion:**

There was no difference in postoperative curve correction or pelvic obliquity between those treated with IOT versus those without during PSF.

**Level of Evidence:**

III.

## Introduction

Neuromuscular scoliosis (NMS) is a challenging condition for patients, caregivers, and physicians. The impairments can disrupt the ability to maintain spinal alignment, often resulting in progressive scoliosis characterized by significant spinal deformity and pelvic obliquity (PO) [1]. This may lead to sitting intolerance, development of pressure ulcers, increased difficulty with nursing care, challenges with transfers and transportation, and restricted pulmonary function [2–7].

The management of NMS is multifaceted and determining the optimal surgical approach for severe, rigid curves among patients with NMS remains the subject of ongoing debate. Various adjunct techniques have been explored, including intraoperative traction (IOT) which utilizes a combination of skull and femoral traction; however, equipoise exists for the optimal approach [8–11].

Despite promising outcomes reported in the literature, existing studies are limited by differences in preoperative curve severity and patient heterogeneity, potentially confounding the outcomes of those studies. As IOT is used at our institution, the purpose of this study was to evaluate the utility of IOT in a matched cohort at a high-volume NMS center. We hypothesized that there would be no significant difference in curve or pelvic obliquity correction in patients with intraoperative traction (IOT) compared to controls (non-IOT).

## Methods

**Fig. 1:**
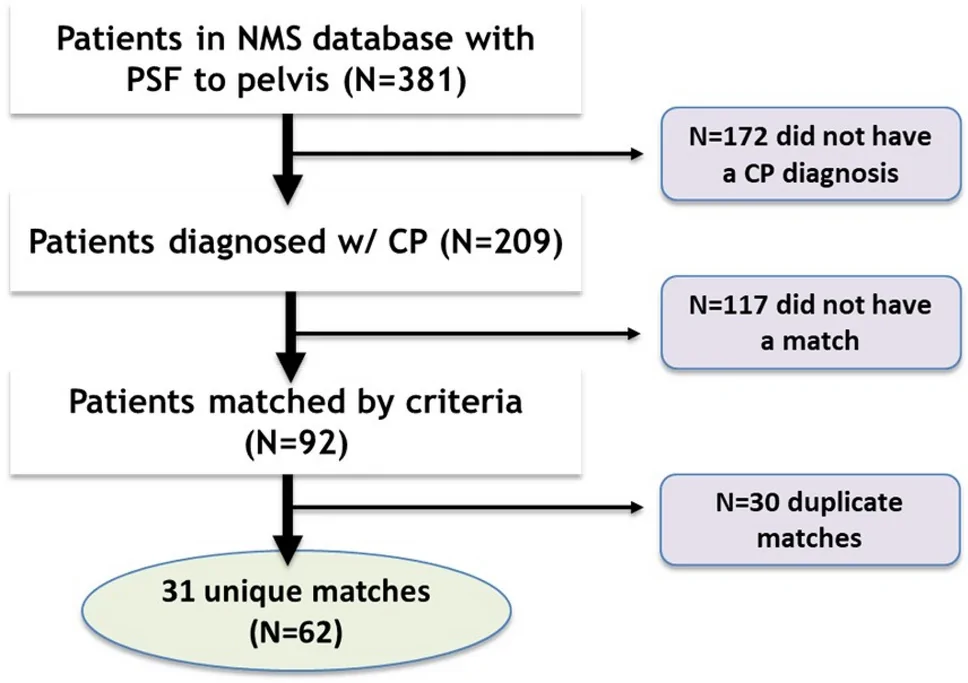
Inclusion/exclusion flowchart

A nested case–control study was performed on a retrospective database of 381 NMS patients aged 6 to 21 years old who underwent posterior spinal fusion (PSF) to the pelvis with 5 treating surgeons at a single large-volume center between January 2005 and August 2021. For the purposes of this study, cases were defined as patients who received IOT during PSF and controls as patients undergoing PSF without IOT. No patients received any form of preoperative traction. Only patients diagnosed with cerebral palsy (CP) and Gross Motor Function Classification System (GFMCS) IV/V were included. Within this group, patients were carefully pair-matched by the following criteria: age within 1-year, preoperative curve magnitude within 10°, preoperative traction curve magnitude within 10°, and flexibility index (FI) within 5%. If a case patient matched with more than one control patient or vice versa, the matched pair with the smallest difference in preoperative curve magnitude was included. The remaining matches were excluded, so that all matched pairs ultimately included in the final analysis would be unique. Figure 1 demonstrates the breakdown of patient selection through inclusion and exclusion criteria. IOT was applied to patients according to the methods previously described by Coughlin et al. [10].

Depending on the distribution of the data, a series of paired *t*-tests or Wilcoxon signed-rank tests were used to analyze continuous variables between case and control groups, including postoperative curve magnitude, percent curve correction, PO, and percent correction of PO. Results from *t*-tests were presented as means ± standard deviation (SD), and results from Wilcoxon signed-rank tests were reported as medians with interquartile range (IQR). Groups were also compared by the number of osteotomies performed during surgery, operative time in minutes (including anesthesia, surgical, and in-room times), estimated blood loss (EBL), intensive care unit (ICU) length of stay, number of levels fused, and body mass index (BMI) using either paired *t* tests or Wilcoxon signed-rank tests. Fisher’s Exact tests were used to compare case and control groups by sex distribution, intraoperative neuromonitoring changes (IONM) signal changes including (Somatosensory Evoked Potentials (SSEPs) and Motor Evoked Potentials (MEPs)), and complication rates and types between groups. Additionally, complications were carefully documented during the 2-year follow-up period and categorized by severity using the Modified Clavien–Dindo-Sink Classification (mCDS), which were also compared between groups using a Fisher’s Exact test [12].

Lastly, a post hoc power (sensitivity) analysis was conducted using the observed paired differences and standard deviations in our primary outcome, degrees of curve correction (SD = 18.17), to estimate the minimum detectable difference given the sample size of *N* = 31 at *α* = 0.05.

## Results

### Preoperative measurements

Thirty-one unique case–control matches were identified for a total of 62 patients. Males made up 53.2% (33/62) of the total cohort and did not differ significantly between the IOT vs. non-IOT groups (17/31 (54.8%) vs. 16/31 (51.6%), *p* > 0.999). Median preoperative curve magnitude was 88° (IQR 80–107°) among the entire cohort and differed by less than 1º between groups. Median age was 14 years for both groups. Traction curve magnitude was 65.8° ± 17.4º overall (65.3 ± 17.5 for IOT vs 66.3 ± 17.6 non-IOT). Average FI was 29.3% overall (29.5% ± 12.7% for IOT vs 29.2% ± 12.6% non-IOT). (Table 1).

**Table 1:** Preoperative measurements between groups

Variable	Cases (IOT, *N* = 31)	Controls (non-IOT, *N* = 31)	*p* value
Age (years)	14 (IQR 12–17)	14 (IQR 13–16)	0.6805
Sex, male (*N*, %)	17 (54.8%)	16 (51.6%)	> 0.999
Curve magnitude (degrees)	88 (IQR 80–106)	88 (IQR 79–107)	0.7512
Traction curve magnitude (degrees)	65.3 ± 17.5	66.3 ± 17.6	0.8176
Flexibility index (degrees)	29.5 ± 12.7	29.2 ± 12.6	0.9205
Pelvic obliquity (degrees)	25 (IQR 19–31)	23 (IQR 11–35)	0.6984

Results are presented as *N*(%) for categorical variables, mean ± SD for variables meeting assumptions of normality, and median (IQR p25–p75) for skew variables

### Intraoperative/surgical parameters

**Fig. 2:**
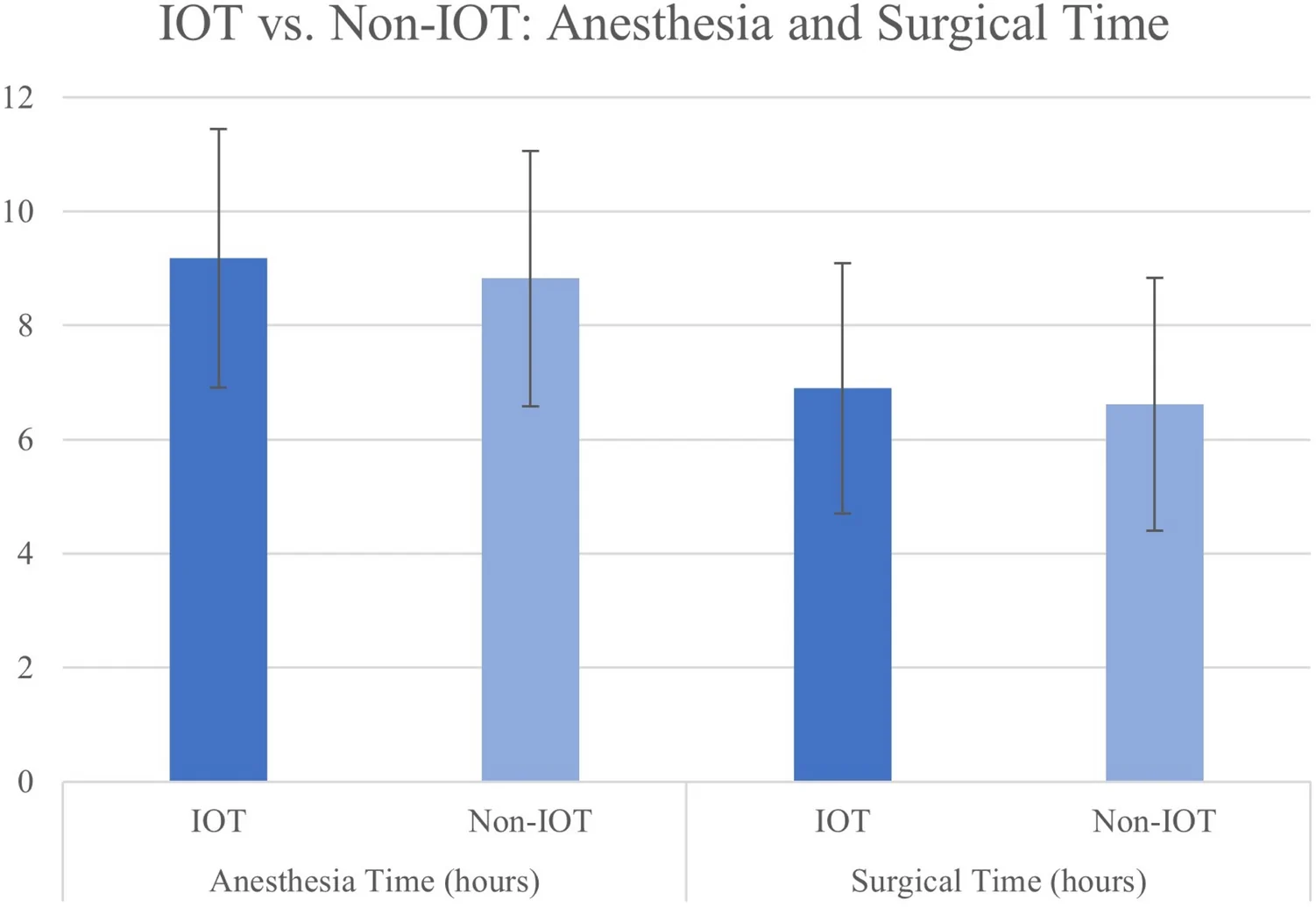
Mean anesthesia time and mean surgical time between the IOT group and the Non-IOT group

**Fig. 3:**
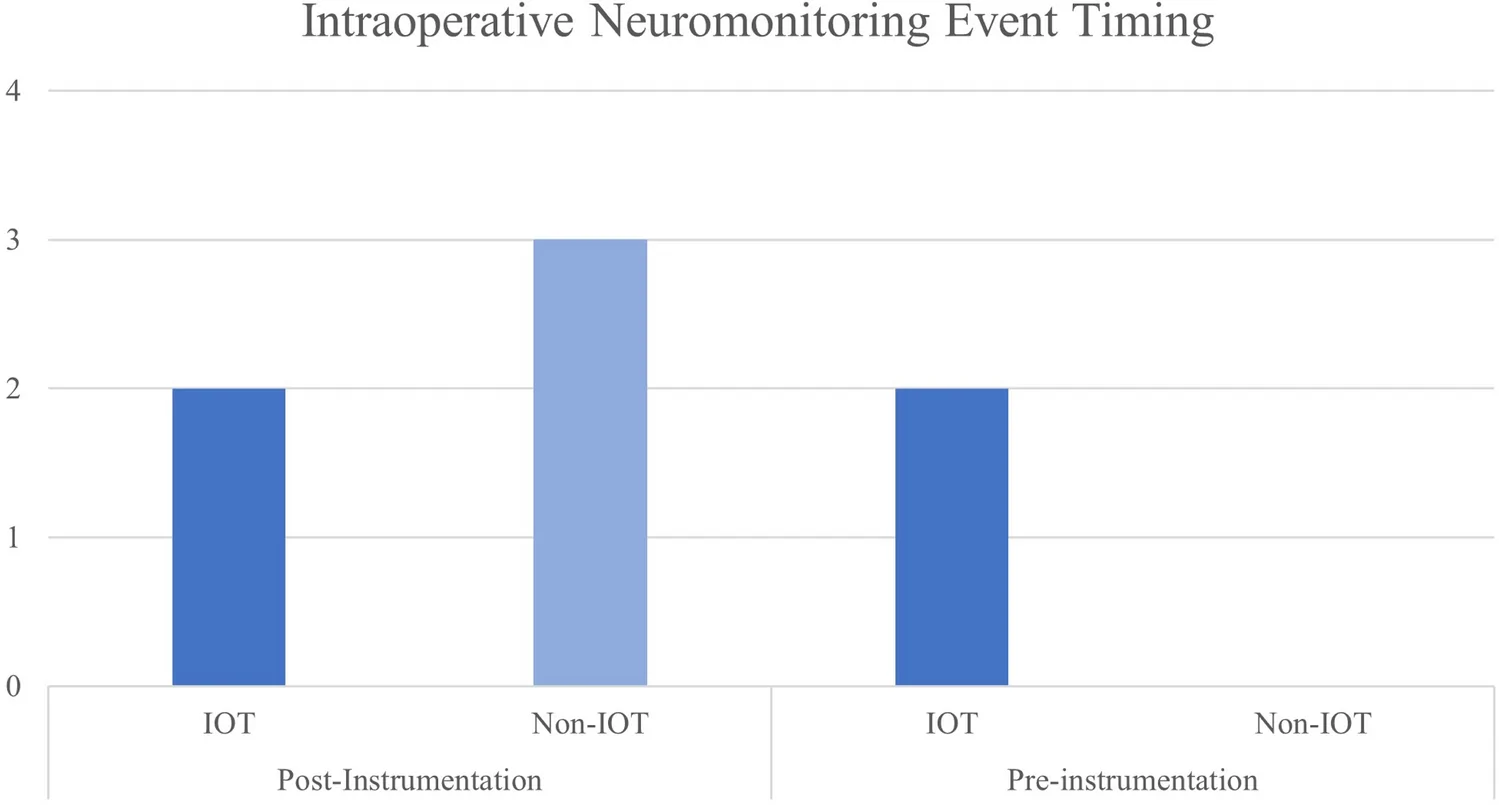
Number of intraoperative neuromonitoring events between the IOT group and Non-IOT group pre-instrumentation and post-instrumentation

Surgical time between groups was similar, with the IOT group averaging 16.9 min longer than the non-IOT group (6.9 ± 2.2 vs 6.6 ± 2.2 h, *p* = 0.6388). Similarly, time under anesthesia (9.2 ± 2.3 vs 8.8 ± 2.2 h, *p* = 0.5585) was slightly longer for those with IOT but did not reach statistical significance. (Fig. 2) Intraoperative EBL was slightly higher among the IOT group (855 (IQR 500–1200) vs 800 (IQR 650–1350), *p* = 0.5980). The number of levels fused was nearly identical between cases (median = 16 levels in both groups; *p* = 0.27). Among those who had osteotomies, the number performed did not differ significantly between the IOT and non-IOT groups (median = 4 vs. 5.5; *p* = 0.44). Implant density was also comparable between groups (median 1.53 vs. 1.61 screws/level; *p* = 0.35). Patients in both the groups experienced similar rates of IONM events (IOT: 4/31 (12.9%) vs. non-IOT: 3/31 (9.7%), *p* > 0.999). (Table 2; Fig. 3).

**Table 2:** Surgical measurements between groups

Variable	Cases (IOT, *N* = 31)	Controls (non-IOT, *N* = 31)	*p* value
Number of osteotomies (median)	5.5 (IQR 3–8)	4 (IQR 3–7)	0.4371
Estimated blood loss (ccs)	855 (IQR 500–1200)	800 (IQR 650–1350)	0.5980
Change in neuromonitoring signals (*N*, %)	4/31 (12.9%)	3/31 (9.7%)	> 0.999
Surgical time (minutes)	413.7 ± 131.4	396.8 ± 132.9	0.6388
Anesthesia time (minutes)	550.6 ± 135.8	529.2 ± 134.7	0.5585
Complications	7/31 (22.6%)	8/31 (25.8%)	> 0.999

Results are presented as *N*(%) for categorical variables, mean ± SD for variables meeting assumptions of normality, and median (IQR p25–p75) for skew variables

### Postoperative outcome measures

**Fig. 4:**
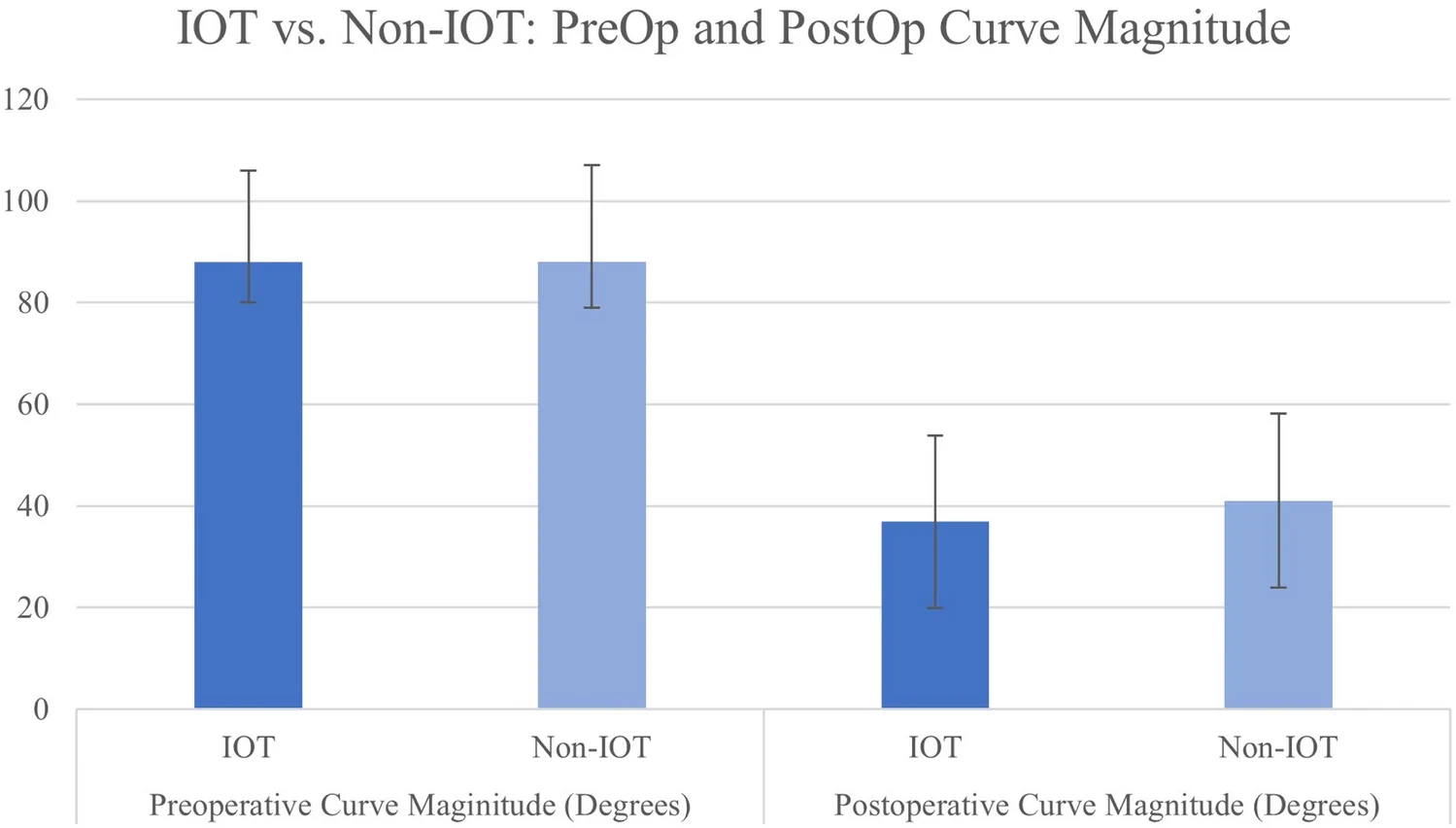
Preoperative and postoperative curve magnitude between the IOT group and Non-IOT group

**Fig. 5:**
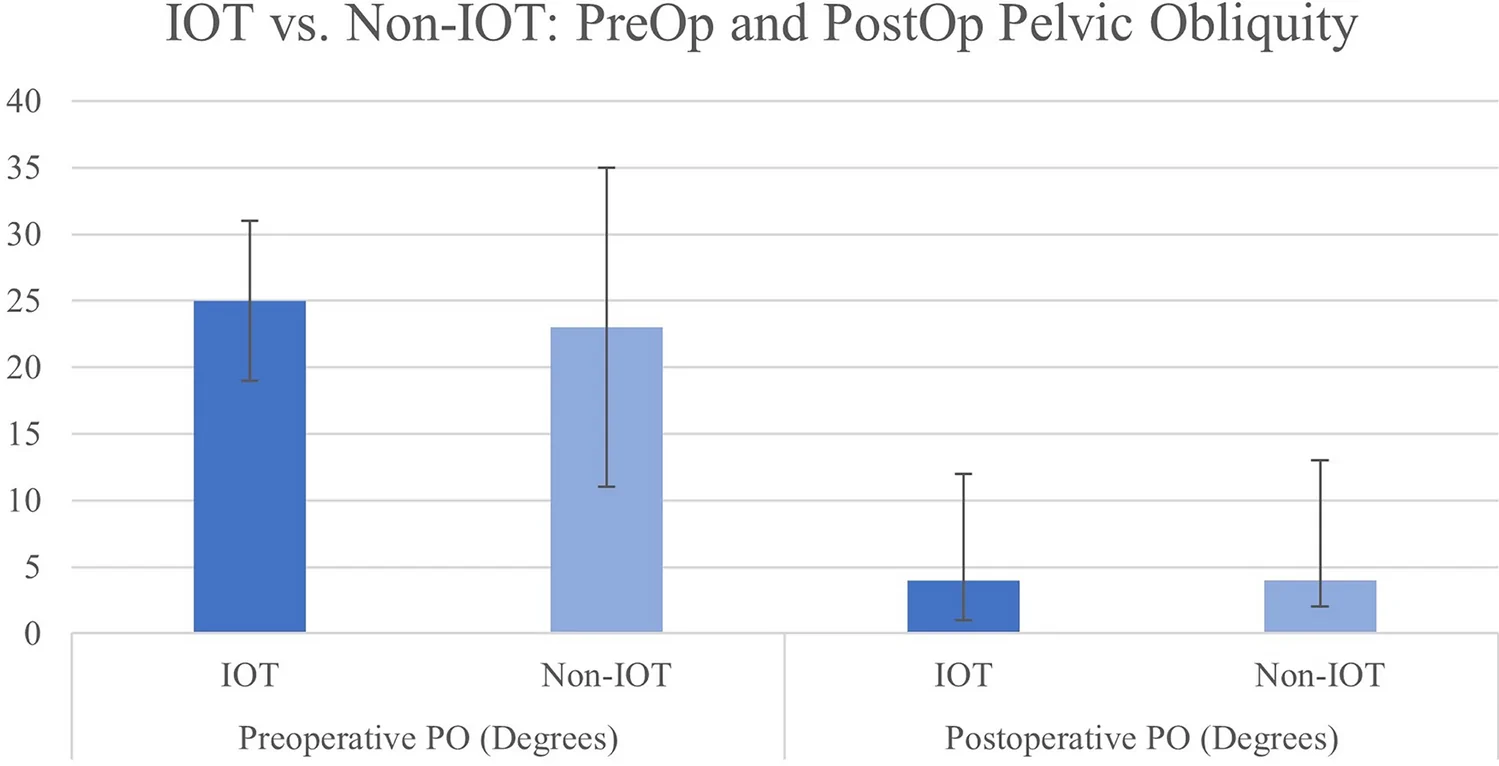
Preoperative and postoperative pelvic obliquity between the IOT group and Non-IOT group

**Fig. 6:**
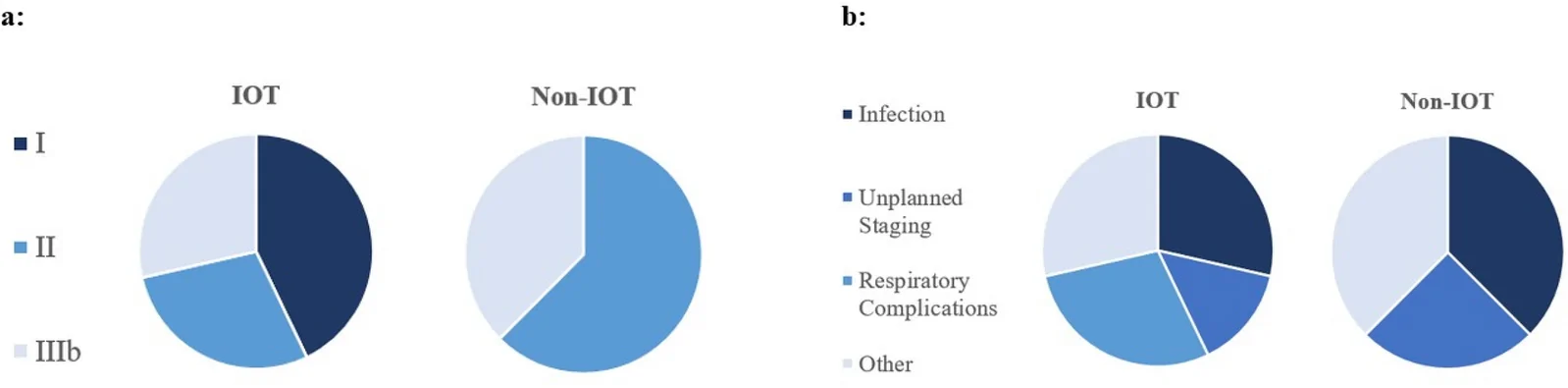
**a** Displays the type of complication that occurred by group, and **b** displays the distribution of complications between groups according to mCDS classification

Residual curve magnitude (36.9 ± 17.0 vs 41.6 ± 17.1, *p* = 0.2775) and percent curve correction (60.1 ± 16.9 vs 56.0 ± 14.3, *p* = 0.3030) were similar between the IOT group and the non-IOT group (Fig. 4). Moreover, postoperative residual PO in degrees (4 (IQR 1–12) vs. 4 (IQR 2–13), *p* = 0.5625) and percent correction of PO (78.3 (IQR 66.7–90.2) vs. 68.3 (IQR 60.2–90.5), *p* = 0.1841) were not significantly different between groups. (Table 3; Fig. 5) There were no differences in complication occurrence (IOT 7/31 (22.6%) vs. non-IOT 8/31 (25.8%) (*p* > 0.999)) or severity according to mCDS classification between groups (*p* = 0.1787). (Table 4; Fig. 6a, b). However, time from surgery to complication onset was longer in the non-IOT group compared to the IOT group (median 30 days [IQR 29–332] vs. median 7 days [IQR 0–22]; *p* = 0.028).

**Table 3:** Postoperative measurements between groups

Variable	Cases (IOT, *N* = 31)	Controls (non-IOT, *N* = 31)	*p* value
Residual curve magnitude (degrees)	36.9 ± 17.0	41.6 ± 17.1	0.2775
Percent curve correction (%)	60.1 ± 16.9	56.0 ± 14.3	0.3030
Residual pelvic obliquity (degrees)	4 (IQR 1–12)	4 (IQR 2–13)	0.5625
Percent pelvic obliquity correction (%)	78.3 (IQR 66.7–90.2)	68.3 (IQR 60.2–90.5)	0.1841

Results are presented as *N*(%) for categorical variables, mean ± SD for variables meeting assumptions of normality, and median (IQR p25–p75) for skew variables

**Table 4:** Complications case (IOT) vs control (non-IOT)

Complication	Case (IOT)	Control (non-IOT)	Total
Infection	2	3	5
Unplanned staging	1	2	3
Respiratory complications	2	0	3
Other	2	3	8
Total	7	8	15
mCDS classification			
I	3	0	3
II	2	5	7
IIIb	2	3	5
Total	7	8	15

*p* = 0.1787

### Sensitivity testing

The post hoc sensitivity analysis demonstrated that the study had 80% power to detect a minimum of 9.6º of curve correction between matched patients with and without intraoperative traction.

## Discussion

The purpose of this study was to evaluate the utility of IOT in a matched cohort at a high-volume NMS center. Contrary to prior literature, we did not find evidence to suggest superior outcomes in patients treated with IOT. Both groups had excellent correction of pelvic obliquity and curve magnitude. Importantly, complication rates were also similar; therefore, this study further establishes the safety of IOT. Our attempts to control for confounding factors netted nearly identical preoperative characteristics between groups in terms of age, sex, preoperative curve magnitude (88º vs. 88º), PO (25º vs. 23º), mean traction curve magnitude (65.3° vs. 66.3°), and FI (29.5°vs. 29.2°). Previous studies were limited by dissimilarities between groups with studies noting 10°–21° differences in curve magnitude and 50–82% differences in PO [8, 13, 14]. In this carefully matched cohort, we found that IOT was a safe adjunctive technique but did not provide a significant benefit in terms of correction.

Advocates for IOT cite its ability to achieve significant correction in both coronal curve magnitude and PO while avoiding increased operative time, hospital time, and complication rates associated with PSF alone, alternative approaches such as halo-gravity traction (HGT), or the more invasive combined anterior/posterior approaches [8, 13–22]. Several studies have demonstrated that PSF with IOT offers comparable or superior coronal correction and significantly improved PO compared to anterior approaches, with additional benefits including reduced operative time, anesthesia duration, estimated blood loss (EBL), postoperative intubation rates, and pulmonary complications, such as pneumonia [13, 14, 21, 23]. Furthermore, while HGT remains a viable option for medically fragile patients whose underlying comorbidities may require gradual correction, IOT offers the advantage of eliminating the burdensome preoperative hospitalization period associated with HGT, with one meta-analysis reporting mean durations of 50.1 days (range 28–79) [24]. This reduction in preoperative length of stay mitigates disruptions to daily life of the patients and their family, decreases financial burden, and lowers the risk of minor complications, such as pin site infections [25, 26].

Existing studies reported mean curve correction of 34°-–68° and correction rates of 38–70% in the IOT groups [8, 11, 13, 14, 20, 21, 23, 27, 28]. A meta-analysis performed by Barik et al. found that the residual curve magnitude was significantly lower in the traction group when compared to the non-traction group (SMD – 0.36, *p* = 0.14) [15]. In studies that included a comparative group without IOT, results ranged from 31° to 80° for final coronal mean correction, 44–66% correction rates, and a mean correction rate difference between groups ranging from 4 to 19% with a trend toward a greater percent correction in the IOT group [8, 13, 14, 20, 23]. In contrast, these differences in mean correction rate were notably nonexistent within the results of our study. Although there was a trend toward slightly improved curve correction percentage (60.1% vs. 56.0%) and residual curve magnitudes for IOT (36.9° vs. 41.6°), these did not achieve significance. Additionally, our residual curve difference of 4.7° could be explained by the accepted measurement error of 5° for Cobb angles and would be deemed irrelevant given failure to surpass this threshold for clinical relevance [29–31]. Based on the results of this study, we have changed our philosophical relationship with traction. Our experience would suggest that to have significant success with traction, the equation must include both traction and time. Therefore, in patients who are deemed candidates for traction, we now favor preoperative halo-gravity traction over IOT.

In our cohort, the residual PO (4°vs. 4°) and PO correction percentage (78.3% vs. 68.3%) were similar among groups. In comparison, the mean PO correction reported in the literature ranged from 10° to 34° with correction rates of 38–87% in the IOT group compared to correction rates of 24–71% for the non-IOT groups [8, 13, 14, 20, 21, 23, 27]. The accepted error rate for PO based on intra/interobserver data is a difference of ± 5° to coincide with Cobb angle acceptable error rates given no previously reported error rates [28]. Furthermore, a cross-sectional study within a healthy population found that slight PO was normal (0°–5.6°, median 2.0°) [32]. Thus, our final PO for both groups was within acceptable error rates for PO and within normal obliquity ranges.

Surgical, anesthesia, and in-room times prove to be more problematic to interpret across multiple studies given the heterogeneity that exists. Meta-analysis data noted trends toward non-clinically significant improved operative times in the IOT group (SMD – 1.09); however, these data are replete with variability within existing studies, such as comparing IOT groups with PSF only to control groups including combined anterior–posterior approaches that inherently required longer operative time needed for dual approaches/dissection, multiple surgical positions, re-draping, etc. [14, 15]. Our results yielded non-statistically significant trends toward increased surgical time (413 vs. 396 min) and time under anesthesia (550 vs. 529 min) in the IOT group, with differences of about 17 min and 21 min, respectively. This difference can potentially be explained by the additional time necessary for traction application. However, our exact time spent applying traction is not included due to unavailability in retrospective documentation.

The incidence of complications (7/31 (22.6%) IOT vs 8/31 (25.8%) non-IOT) and complication severity by mCDS classification was not different between groups (*p* = 0.1787) [12]. This is comparable to the overall NMS complication rate previously reported (17.9–81%) and for IOT reported as 0–54.5% [8, 13, 14, 19, 21, 33–36]. There were 2 mCDS IIIb complications in the IOT group with an unplanned staging to accommodate a complex wound closure flap procedure and a deep infection. However, the non-IOT group had 3 mCDS IIIb complications with 2 unplanned stagings for IONM events and a deep infection. All mCDS IIIb complications required an additional surgery. Our reoperation rate of 6.4% (2/31) for IOT and non-IOT 9.7% (3/31) was less than reported previously for both overall NMS cases 16% and for studies with IOT groups 8.0–12.2% [8, 20, 27, 33]. Our overall infection rate of 6.4% (2/31) for IOT vs. non-IOT 9.7% (3/31) was comparable to the overall infection rates previously seen in both overall NMS cases 3.6–8.7% and groups with IOT 0–18.1% [8, 13, 14, 21, 27, 33, 36–38]. Each group in our cohort had 1 deep infection for a 3.2% (1/31) for both groups, which was also comparable to the previously reported overall NMS and IOT group deep infection rates of 2.1–11.9% and 0–7.3%, respectively [8, 13, 21, 27, 33–39]. These results align with previous studies that have shown IOT to be safe in NMS patients with non-statistically significant lower complication rates when compared to controls without IOT [8, 13, 15, 19]. Although complication type and severity were similar between groups, there were timing differences between groups, with time from surgery to complication was longer among those in the non-IOT group. We found a minor increase in EBL among our IOT group that was not significant (*p* = 0.60). Other studies, including a 2023 meta-analysis, have shown a clinically insignificant trend toward decreased EBL with IOT (SMD – 0.86) [15]. Full complications are reported in Table 4, and a breakdown of complications by type and mCDS Classification is presented in Fig. 6A, B, respectively.

Previous studies have shown no significant difference in IONM changes between IOT and non-IOT groups. A study by Tondevold et al. reported IONM events in 24% of patients in the IOT group and 33% of patients in the non-IOT group, while Padhye et al. documented one single occurrence of IONM changes in the non-IOT group and none among those in the IOT group [8, 40]. Moreover, Berends et al. published a 13.6% IOT rate overall in their cohort with the highest rate in NMS patients at 20.6% [16]. In our study, the rate of IONM signal changes was similar between groups, with the IOT group having changes in 4/31 (12.9%) vs. 3/31 (9.7%) for non-IOT. In the non-IOT group, all three IONM events occurred during instrumentation, whereas in the IOT group, 2/4 occurred post-traction/pre-instrumentation and the other two occurred during instrumentation. Two out of three non-IOT patients with neuromonitoring changes required unplanned staging with return to baseline after corticosteroid initiation in one and removal of the rods in another. For the two patients in the IOT group who lost signals post-traction/pre-instrumentation, they had signals return after removing traction alone. Overall, 1/7 (14.3%) IONM events resolved with increasing mean arterial pressures (MAPs) alone and 4/7 (57.1%) resolved with increasing MAPs in conjunction with other interventions (removing traction weight or reducing the correction). This is similar to previous studies that reported 20% improvement with increasing MAPs alone and 60% with increasing MAPs in conjunction with other interventions [41]. All neuromonitoring changes were transient and reversed without long-term neurological sequelae.

The strength of our study is that it is a nested case–control study performed at a high-volume, NMS center by 5 surgeons. Contrary to existing literature, our study has decreased variability among groups with diagnosis, sex, age, curve magnitude, traction curve magnitude, PO, and FI allowing for more controlled comparison of IOT and non-IOT groups. This is especially notable with our study’s preoperative curve magnitudes, which are identical, compared to some studies with 10°–20° differences between groups [13, 40]. NMS has a wide variety of underlying diagnoses, each with its own subtleties, that create confounding factors between groups and prevent complete generalizability. Therefore, focusing strictly on patients with an underlying diagnosis of CP for inclusion limited confounding factors commonly found within the current literature, including studies that aggregate both AIS and NMS cases, or combined patients of multiple neuromuscular etiologies [13, 27, 40].

Despite the strengths of this study, it is not without limitations. The first limitation is the retrospective nature of this study. Additionally, the controlled pair matching from a single-center database led to a relatively small sample size. Furthermore, generalizability is limited given this is a single-center study with similar surgeon philosophies for patient selection and traction protocols. IOT was utilized based on surgeon discretion in this cohort. As this was a retrospective study, we do not have data on the factors that informed surgeon decision to utilize IOT. However, the surgeons were equally represented in both the IOT and non-IOT groups*.* It is also worth noting that there was heterogeneity in femoral traction methodology within the IOT group, as there was a shift of philosophy at our institution around 2011. This includes 4 (12.9%) cases from 2010 to 2011 utilizing skeletal traction with Kirschner wires or Steinmann pins in the distal femur, while the remaining 27 (87.1%) cases (all taking place in 2012 or later) received skin traction with tape along the patient’s thigh and buttock region. This method has been described in detail by Coughlin et al. [10].

A post hoc sensitivity analysis demonstrated that the sample size (*N* = 31 pairs) provided 80% power to detect a difference of 9.6° in curve correction between groups. Given that the intra- and interobserver variability in Cobb angle measurement has been reported at between 4 and 8° in the literature, a detectable difference of 9.6° exceeds the range of measurement error, suggesting that any clinically meaningful differences in curve correction between groups would have likely been detected with the available sample size [31, 42–44].

IOT is a safe adjunct to consider for large curves in NMS; however, in a carefully matched cohort of NMS patients, IOT was not associated with improved correction of coronal curve magnitude or PO contrary to existing published data. Further study implementing prospective and randomized multicenter efforts with standardized protocols is needed to investigate the optimal approach to achieve correction of NMS curves.

## Data Availability

The data used in this study are available upon request.
